# Immunochromatography for the diagnosis of *Mycoplasma pneumoniae* infection: A systematic review and meta-analysis

**DOI:** 10.1371/journal.pone.0230338

**Published:** 2020-03-17

**Authors:** Seo Hee Yoon, In Kyung Min, Jong Gyun Ahn

**Affiliations:** 1 Department of Pediatrics, Severance Children’s Hospital, Yonsei University College of Medicine, Seoul, Korea; 2 Biostatistics Collaboration Unit, Department of Biomedical Systems Informatics, Yonsei University College of Medicine, Seoul, Korea; Instituto Butantan, BRAZIL

## Abstract

The aim of this study was to evaluate the diagnostic performance of immunochromatographic tests (ICTs) for the detection of *Mycoplasma pneumoniae*. Medline/Pubmed, Embase, the Cochrane Library, and ISI Web of Science were searched through June 12, 2019 for relevant studies that used ICTs for the detection of *M*. *pneumoniae* infection with polymerase chain reaction (PCR) or microbial culturing as reference standards. Pooled diagnostic accuracy with 95% confidence interval (CI) was calculated using a bivariate random effects model. We also constructed summary receiver operating characteristic curves and calculated the area under the curve (AUC). Statistical heterogeneity was evaluated by χ^2^ test or Cochrane’s Q test. Thirteen studies including 2,235 samples were included in the meta-analysis. The pooled sensitivity and specificity for diagnosing *M*. *pneumoniae* infection were 0.70 (95% CI: 0.59–0.79) and 0.92 (95% CI: 0.87–0.95), respectively. The positive likelihood ratio (LR) was 8.94 (95% CI: 4.90–14.80), negative LR 0.33 (95% CI: 0.22–0.46), diagnostic odds ratio 29.20 (95% CI: 10.70–64.20), and AUC 0.904. In subgroup analysis, ICTs demonstrated similar pooled sensitivities and specificities in populations of children only and mixed populations (children + adults). Specimens obtained from oropharyngeal swabs exhibited a higher sensitivity and specificity than those of nasopharyngeal swab. Moreover, pooled estimates of sensitivity and accuracy for studies using PCR as a reference standard were higher than those using culture. The pooled sensitivity and specificity of Ribotest Mycoplasma^®^, the commercial kit most commonly used in the included studies, were 0.66 and 0.89, respectively. Overall, ICT is a rapid user-friendly method for diagnosing *M*. *pneumoniae* infection with moderate sensitivity, high specificity, and high accuracy. This suggests that ICT may be useful in the diagnostic workup of *M*. *pneumoniae* infection; however, additional studies are needed for evaluating the potential impact of ICT in clinical practice.

## Introduction

*Mycoplasma pneumoniae* is an important cause of respiratory tract infection (RTI) in school-age children and young adults [1–4]. *M*. *pneumoniae* is responsible for approximately 10–40% of community-acquired pneumonia (CAP) cases [3, 5], rising to 70% in closed populations during epidemics [6–8]. *M*. *pneumoniae* infection is primarily known to present with a mild clinical course [6]; however, 3–4% of those are reported to develop into fulminant pneumonia with hypoxia [9, 10]. Extrapulmonary complications, primarily being central nervous system complications, may also occur in approximately 25% of *M*. *pneumoniae*-infected individuals [1, 11].

*M*. *pneumoniae* lacks a cell wall, and therefore, β-lactam antibiotics, which are active against most respiratory bacterial pathogens for RTI in children, are ineffective against *M*. *pneumoniae* [12]. A prompt and precise diagnosis of *M*. *pneumoniae* infection leads to the use of appropriate antibiotics. However, it is difficult to distinguish *M*. *pneumoniae* from other causative microorganisms of RTI early during the clinical course based on patient history, symptoms, physical examination, or a chest radiograph. Therefore, laboratory confirmation of the microorganism is crucial for planning the appropriate management [13–16].

While microbial culturing has been a gold standard for *M*. *pneumoniae* diagnosis, *M*. *pneumoniae* are fastidious and cultivation may require weeks for growth. Therefore, culturing is not routinely performed in clinical practice [17]. Serology is a more convenient and widely used method than culturing. A single high titer of *M*. *pneumoniae*-specific antibody is indicative of a recent infection; however, false-negative test results often occur early in the course of illness [17, 18]. An increase in the *M*. *pneumoniae*-specific IgG titer ≥ 4-fold during acute and convalescent phases of the clinical course also implies recent infection [19]; however, this is impractical in clinical practice as it requires 2–4 weeks of monitoring [17, 18]. ImmunoCard Mycoplasma (Meridian Bioscience, Cincinnati, OH, USA), a 10-min card-based enzyme-linked immunosorbent assay to detect *M*. *pneumoniae* IgM antibodies, has been developed and is commercially available [20, 21]. However, ImmunoCard Mycoplasma is an assay for IgM only and can exhibit false-positive results for an extended period of time after *M*. *pneumoniae* infection as *M*. *pneumoniae* IgM antibodies may persist for several months [21, 22]. Polymerase chain reaction (PCR) analysis is highly sensitive and currently used as a reference diagnostic method for *M*. *pneumoniae* detection; however, it requires complex and time-consuming sample pretreatment, skilled technical ability, and expensive equipment [23, 24].

Recently, several techniques for the rapid diagnosis of *M*. *pneumoniae* have been developed, including loop-mediated isothermal amplification (LAMP) [25, 26] and the immunochromatographic test (ICT) [27–30]. LAMP is a technique in which DNA is amplified under isothermal conditions within one hour [31, 32]. Although LAMP shows high sensitivity and specificity in the diagnosis of infectious diseases [33], it requires specific equipment for DNA amplification. In addition, an isolated room and a closed reaction system are recommended owing to unintended carryover contamination that may lead to false positives [34, 35]. Therefore, LAMP is not thought to be practical for use in primary care settings [36, 37].

The immunochromatographic test (ICT), often referred to as a lateral-flow assay, is a popular application of enzyme-immunoassays that utilize antigen and antibody properties as a sample passes along a membrane [38–41]. ICT has several positive qualities, including that it is simple and easy to perform, has a rapid assay time, exhibits long-term stability regardless of climate, is inexpensive, and is an instrument-free diagnostic test [42]. Moreover, results can be observed with the naked eye within 10–15 min [28, 29, 43, 44]. Recently, the diagnosis of CAP has been facilitated by the use of ICT-based urinary antigen tests for *Streptococcus pneumoniae* and *Legionella pneumophila* serogroup-1 [45, 46]. ICT targeting of *M*. *pneumoniae* antigen (e.g. ribosomal protein L7/L12) has also been developed and is commercially available [30, 36, 47–50]. However, the studies that have evaluated the performance characteristics of ICT for the detection of *M*. *pneumoniae* have not currently been systematically reviewed or integrated. Therefore, the aim of this systematic review and meta-analysis was to integrate and assess the evidence for the diagnostic accuracy of ICT for *M*. *pneumoniae* infection.

## Materials and methods

This review was performed in accordance with the Preferred Reporting Items for Systematic Reviews and Meta-Analyses Statement (PRISMA) [51]. The protocol has been registered with Prospero: International prospective register of systematic reviews (registration number CRD42019140809).

### Literature search

We searched on Medline/Pubmed, Embase, the Cochrane Library, and ISI Web of Science using the keywords *Mycoplasma pneumoniae*, immunochromatography and lateral flow assay. The search strategy included “*Mycoplasma pneumoniae* AND immunochromatography OR lateral flow assay” (S1 Search strategy). The search was executed on June 12, 2019. Additional studies were identified by examining the reference lists of the relevant articles. No language restrictions were applied. As the current study was based on a systematic review of previously published studies, institutional review board approval and patient consent were not necessary. This research received no specific grant from any funding.

### Eligibility criteria

Studies were considered eligible if they assessed the accuracy of ICT for the diagnosis of *M*. *pneumoniae* infection and were detailed enough to allow the construction of a 2 × 2 table. We defined ICT as any assay identifying *M*. *pneumoniae* antigens in human respiratory specimens using ICT formats. Studies using PCR or microbial cultures as reference standards were eligible for inclusion in the current study. *In vitro* and *in vivo* animal studies were excluded. Editorials, letters to the editors, and conference abstracts were also excluded.

### Study selection and data extraction

Two reviewers (SHY and JGY) independently screened the titles and abstracts for potential relevance and conducted full-text reviews of the selected publications. Any disagreements were resolved by a third reviewer (IKM) following a discussion with all three reviewers. Author names, country of origin for the study, publication year, study design, study period, age distribution of the study population (children were defined as ≤ 18 years of age), participant gender, index test assay, index test target, index test company, reference standard, type of specimen, sample size, and data regarding true positive, false positive, true negative, and false negative were extracted. If studies consisted of multiple groups, each group was treated as a single study. If there was insufficient information to construct the 2 × 2 table, we attempted to contact the corresponding authors by e-mail.

### Quality assessment

The validity of the included studies was assessed using the Quality Assessment of Diagnostic Accuracy Studies 2 (QUADAS-2) tool [52]. QUADAS-2 evaluates the risk of bias and the applicability of diagnostic accuracy studies, which consists of four key domains (patient selection, index test, reference standard, and flow and timing). Each domain was assessed in terms of risk of bias and the first three domains with respect to concerns regarding applicability.

### Statistical analysis

Summary estimates of sensitivity and specificity along with 95% confidence intervals were calculated based on a bivariate random effects model [53]. From the pooled estimates, we derived the diagnostic odds ratio (DOR), positive LR, negative LR, and 95% CI [54].

Summary receiver operating characteristic (SROC) curves and the area under the curve (AUC) obtained from the fitted bivariate random effects model were used to summarize the overall test performance. SROC curves were plotted with the confidence region and prediction region. Heterogeneity of sensitivity and specificity was assessed by visual inspection of forest plots and by χ^2^ test analysis (p < 0.05 indicated significant heterogeneity). If heterogeneity between studies existed, a bivariate random effects model was adopted [55–57]. A fixed value (0.5) was added as a continuity correction to all cells for studies with zero values.

We planned subgroup analyses prior to starting the evaluations as heterogeneity among the studies was expected. The following variables were expected to be possible sources of heterogeneity: age of the population (children, adult, mixed), type of index test and type of specimen, reference standard used, and blinding procedures. Potential publication bias was visually assessed using a funnel plot [58, 59]. The statistical significance of publication bias was tested using the Egger’s test [60]. R package, version 3.6.0 (The R Foundation for Statistical Computing, Vienna, Austria), was used for all statistical analyses.

## Results

### Characteristics of the included studies

**Fig 1:**
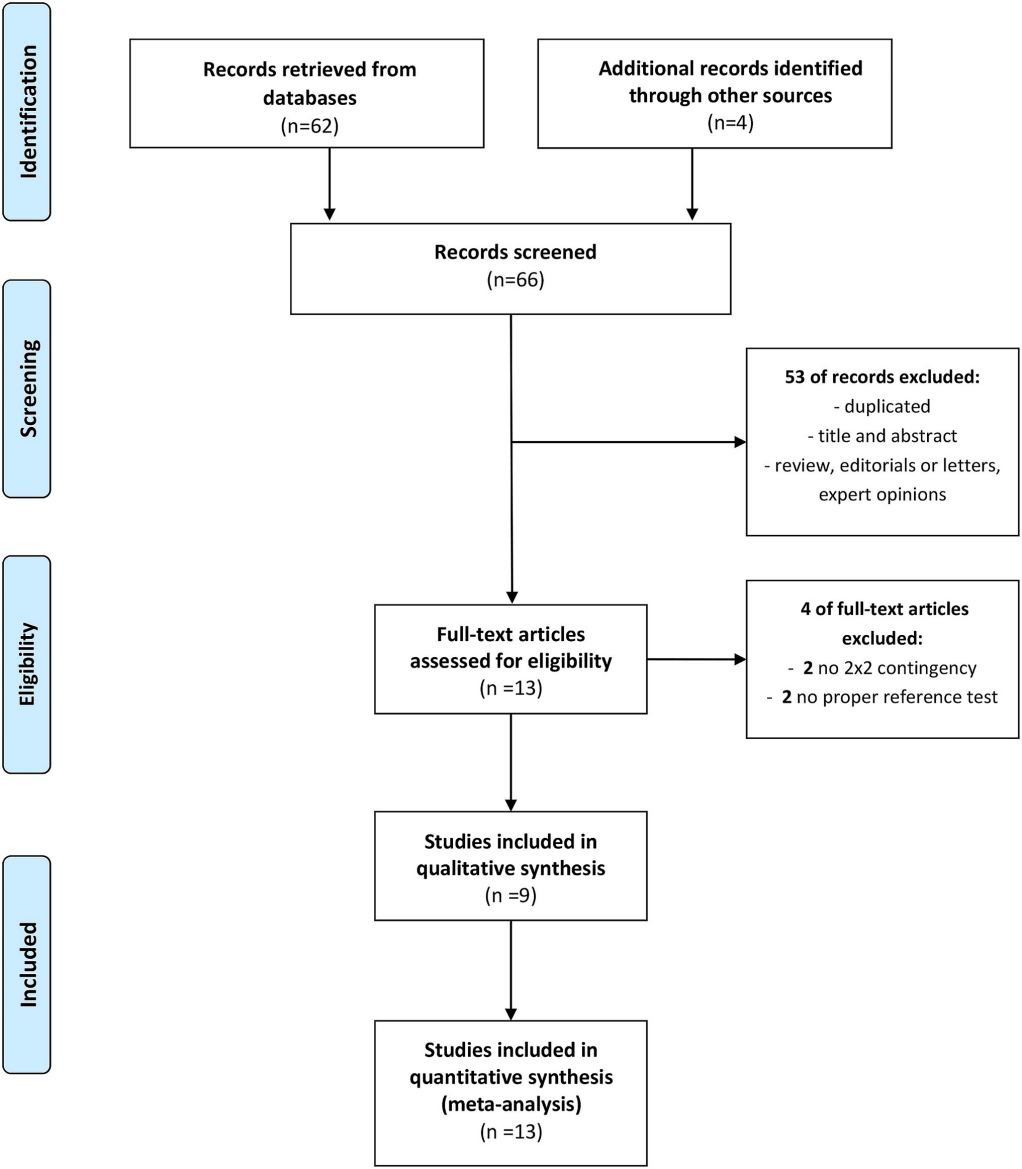
Preferred Reporting Items for Systematic Reviews and Meta-Analyses (PRISMA) flow chart of the selection process used for eligible studies.

Totally, 66 articles were retrieved. After the removal of duplicate articles and exclusion of studies based on titles and abstracts, 13 articles remained for full text review. Four of the 13 studies were excluded as two studies did not provide sufficient data for generation of a 2 × 2 contingency table and the other two studies did not use PCR or microbial cultures as reference standards. Of the nine studies not excluded, several evaluated more than one reference method [28] and different patient groups [48], brand of index test [36], and type of specimen [49]. Each dataset from these studies was considered separately. Therefore, 13 studies (11 in English, 1 in Korean, 1 Japanese) [28–30, 36, 47–50, 61] were ultimately included for quantitative data synthesis and meta-analysis (Fig 1).

The data sets were extracted from the 13 articles and consisted of 2,235 samples. Descriptive characteristics of the studies are summarized in Table 1. All the studies were conducted in Asia (2 in China [29, 61], 10 in Japan [28, 30, 36, 47–49], and 1 in Korea [50]). Seven studies (53.8%) included both adults and children [28, 36, 47, 48] while the remaining six studies (46.2%) included only children [29, 30, 49, 50, 61]. No studies specifically evaluated adult populations (≥ 18 years of age). Among the ICTs used in the studies included in our meta-analysis, Ribotest Mycoplasma^®^ (Asahi Kasei Pharma Co., Tokyo, Japan) was the most frequently assessed with eight (57.6%) of the studies evaluating this ICT [30, 36, 47–50]. PCR was used as the reference standard in 11 (84.6%) of the studies [28–30, 36, 47–49, 61] and microbial culturing was used in two (15.4%) of the studies [28, 50]. No studies described the duration of symptoms prior to testing. The specific age ranges of enrolled patients and gender proportions are summarized in S1 Table.

**Table 1:** Characteristics of the studies included in the meta-analysis.

Year, Author	Country	Study periods	Age	Specimen	Patients	Index test assay	Index test target	Company	Reference standard	MP confirmed/non-MP confirmed (n)
2015, Li	China	Feb 2014 to Aug 2014	Children*	OP swab + sputum	pneumonia + suspected MP infection	Colloidal gold-based IC assay	MP membrane protein P1	In-house ICT	PCR	78/224
2015, Miyashita	Japan	Nov 2013 to Oct 2014	children + adult	NP swab	CAP	Ribotest Mycoplasma^®^	MP L7/L12 ribosomal protein	Asahi Kasei Pharma, Tokyo, Japan	PCR	8/110
2015, Yamazaki	Japan	Sep 2012 to Mar 2013	children	NP swab	pneumonia or bronchitis	Ribotest Mycoplasma^®^	MP L7/L12 ribosomal protein	Asahi Kasei Pharma, Tokyo, Japan	PCR	85/127
2016, Miyashita-1	Japan	May 2015 to Aug 2015	children + adult	NP swab	RTI	Ribotest Mycoplasma^®^	MP L7/L12 ribosomal protein	Asahi Kasei Pharma, Tokyo, Japan	PCR	46/355
2016, Miyashita-2	Japan	May 2015 to Aug 2015	children + adult	NP swab	CAP	Ribotest Mycoplasma^®^	MP L7/L12 ribosomal protein	Asahi Kasei Pharma, Tokyo, Japan	PCR	8/60
2016, Sano-1	Japan	−	children + adult	pharyngeal swab^§^	RTI	Mycoplasma RP-L7/L12 ICT	MP L7/L12 ribosomal protein	In-house ICT	PCR	33/143
2016, Sano-2	Japan	−	children + adult	pharyngeal swab^§^	RTI	Mycoplasma RP-L7/L12 ICT	MP L7/L12 ribosomal protein	In-house ICT	culture	35/141
2017, Kakuya-1	Japan	Dec 2015 to Aug 2016	children	NP swab	community-acquired lower RTI	Ribotest Mycoplasma^®^	MP L7/L12 ribosomal protein	Asahi Kasei Pharma, Tokyo, Japan	PCR	15/43
2017, Kakuya-2	Japan	Dec 2015 to Aug 2016	children	OP swab	community-acquired lower RTI	Ribotest Mycoplasma^®^	MP L7/L12 ribosomal protein	Asahi Kasei Pharma, Tokyo, Japan	PCR	15/43
2017, Song	China	Dec 2016 to Jan 2017	children	OP swab	pneumonia	SWCNT/CGIC strip	MP membrane protein P1	In-house ICT	PCR	97/40
2018, Namkoong-1	Japan	Dec 2015 to Dec 2016	children + adult	OP swab	clinically suspected MP infection	SAI system^¶^	MP antigen	Mizuho Medy, Saga, Japan or Fujifilm, Kanagawa, Japan	PCR	73/84
2018, Namkoong-2	Japan	Dec 2015 to Dec 2016	children + adult	OP swab	clinically suspected MP infection	Ribotest Mycoplasma^®^	MP L7/L12 ribosomal protein	Asahi Kasei Pharma, Tokyo, Japan	PCR	73/84
2019, Yang	Korea	Aug 2010 to Aug 2018	children	NP aspirates	lower RTI	Ribotest Mycoplasma^®^	MP L7/L12 ribosomal protein	Asahi Kasei Pharma, Tokyo, Japan	culture	119/96

CAP, community acquired pneumonia; MP, *Mycoplasma pneumoniae*; PCR, polymerase chain reaction; RTI, respiratory tract infection; SWCNT/CGIC, single-walled carbon nanotubes coupled with the colloidal gold-monoclonal antibody immunochromatographic strips; ICT, immunochromatographic test; NP, naso-pharyngeal; OP, oropharyngeal; SAI, silver amplification immunochromatography

^−^: Not given.

* Children and adults were defined as younger and older than 18 years of age, respectively.

^§^ Authors did not provide details regarding the source of the swabs (nasopharyngeal or oropharyngeal).

^¶^ The SAI system consists of a Quick Chaser^®^ Auto Myco (Mizuho Medy, Saga, Japan) or FUJI DRI-CHEM IMMUNO AG

Cartridge Myco (Fujifilm, Kanagawa, Japan) combined with an analyzer Quick Chaser Immuno Reader (Mizuho Medy,

Saga, Japan) or FUJI DRI-CHEM IMMUNO AG1 (Fujifilm, Kanagawa, Japan).

### Quality assessment

**Fig 2:**
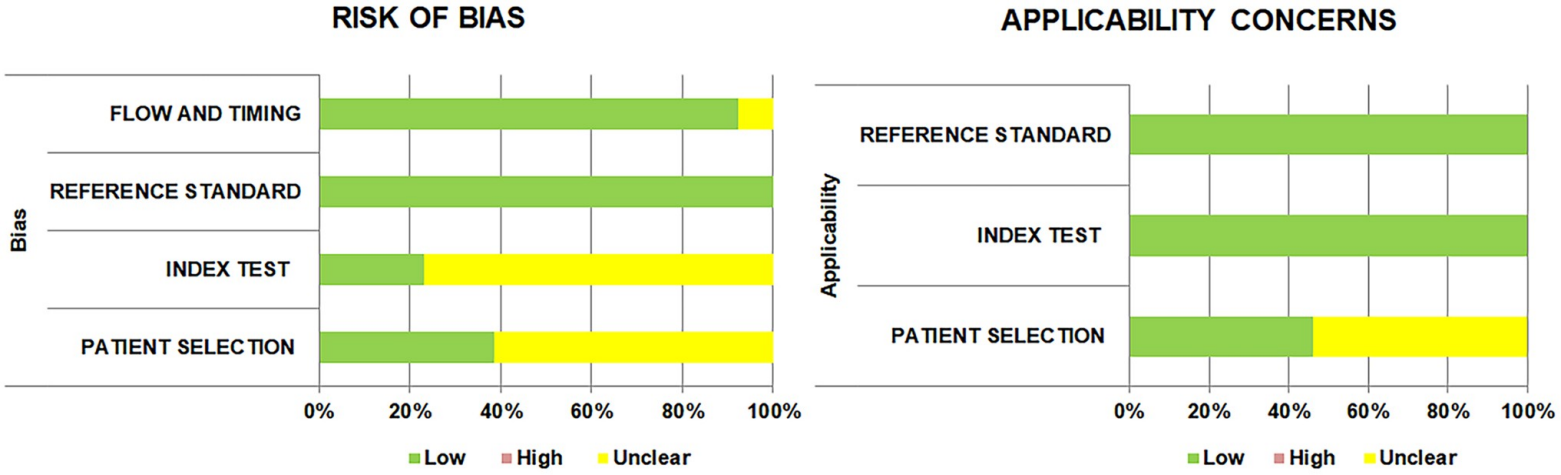
Quality assessment of the diagnostic accuracy studies-2 (QUADAS-2).

Results of QUADAS-2 assessment for evaluating the quality of the studies are shown in Fig 2 and S2 Table. With respect to the risk of bias, in the patient selection domain, 61.5% of the studies were considered “unclear” risk of bias as they failed to specify the methods used for enrollment of the patients, whether consecutive or random. The remaining studies were classified as “low” risk of bias. In the index test domain, most of the studies (76.9%) were classified as “unclear” risk of bias since the authors did not report the index test, did not clarify whether the ICT results were identified without knowledge of the results of the reference standard [62, 63]. However, if index test results were interpreted with dedicated ICT readers, the studies were judged to be at low risk of bias. In the reference standard domain, all studies were at “low” risk of bias because PCR and culturing were regarded as being objective methods, regardless of whether they were interpreted without knowledge of the index test results. In the flow and timing domain, most studies (12/13, 92.3%) were at “low” risk of bias. Applicability was of low concern for all the studies in the index and reference standard domain. The patient selection domain was assessed to be an “unclear” concern for seven of the studies (53.8%) as they enrolled only lower RTI patients.

### Overall accuracy of IC

**Fig 3:**
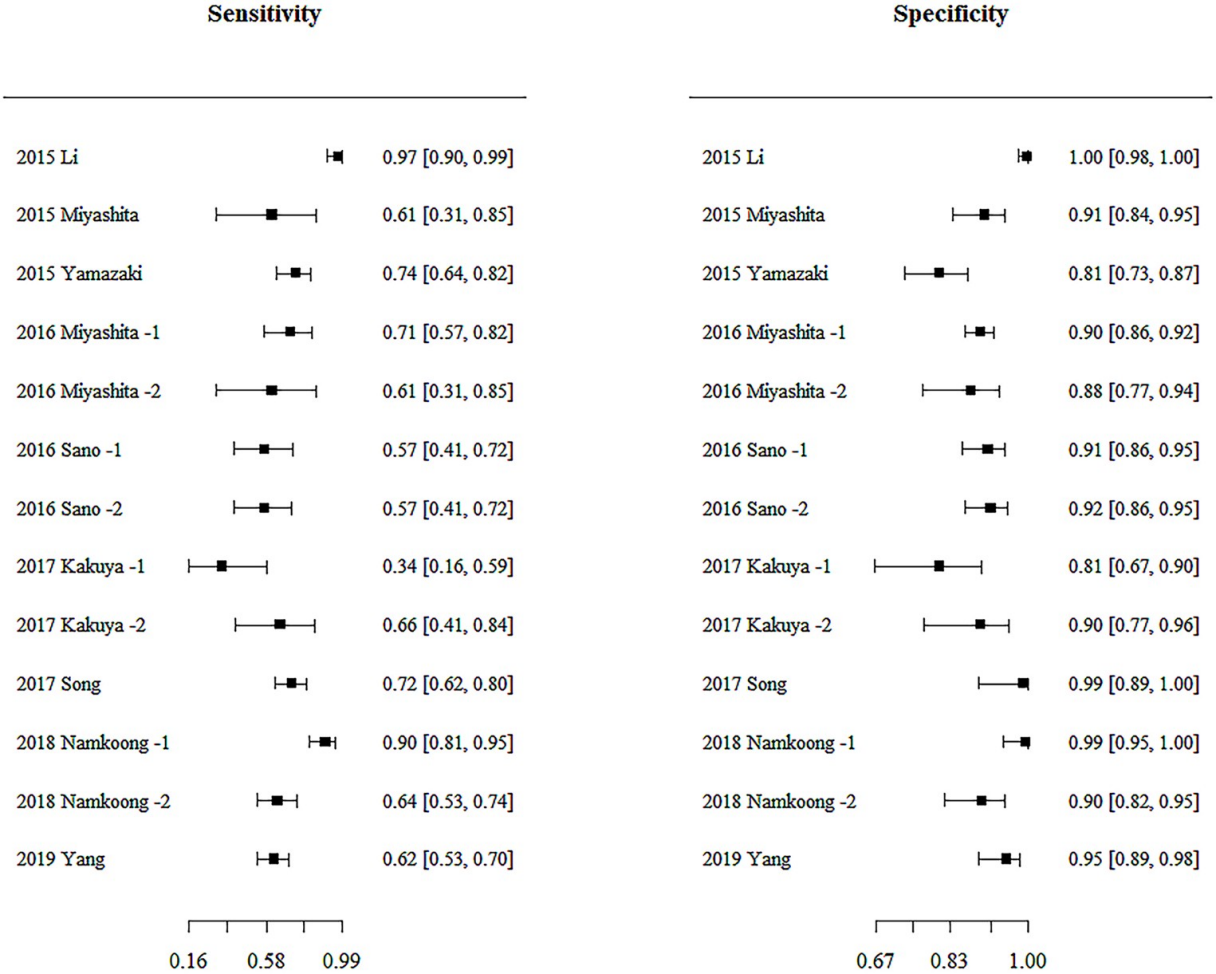
Coupled forest plots of the sensitivity and specificity of immunochromatographic tests for diagnosing *Mycoplasma pneumoniae* infection. The studies are indicated by year and author name. The numbers are pooled estimates with 95% confidence interval (CI) in brackets. Horizontal lines indicate 95% CIs.

The sensitivity and specificity of each study included in the analysis are shown in the form of a forest plot in Fig 3. Significant heterogeneity between studies was noted in terms of sensitivity (χ^2^ = 63.75; p < 0.0001) and specificity (χ^2^ = 60.62; p < 0.0001). Taking into account the statistical heterogeneity, a meta-analysis was performed using a bivariate random effects model. Funnel plot asymmetry (p = 0.0001 from Egger’s test) revealed the existence of publication bias among the included studies (S1 Fig).

**Fig 4:**
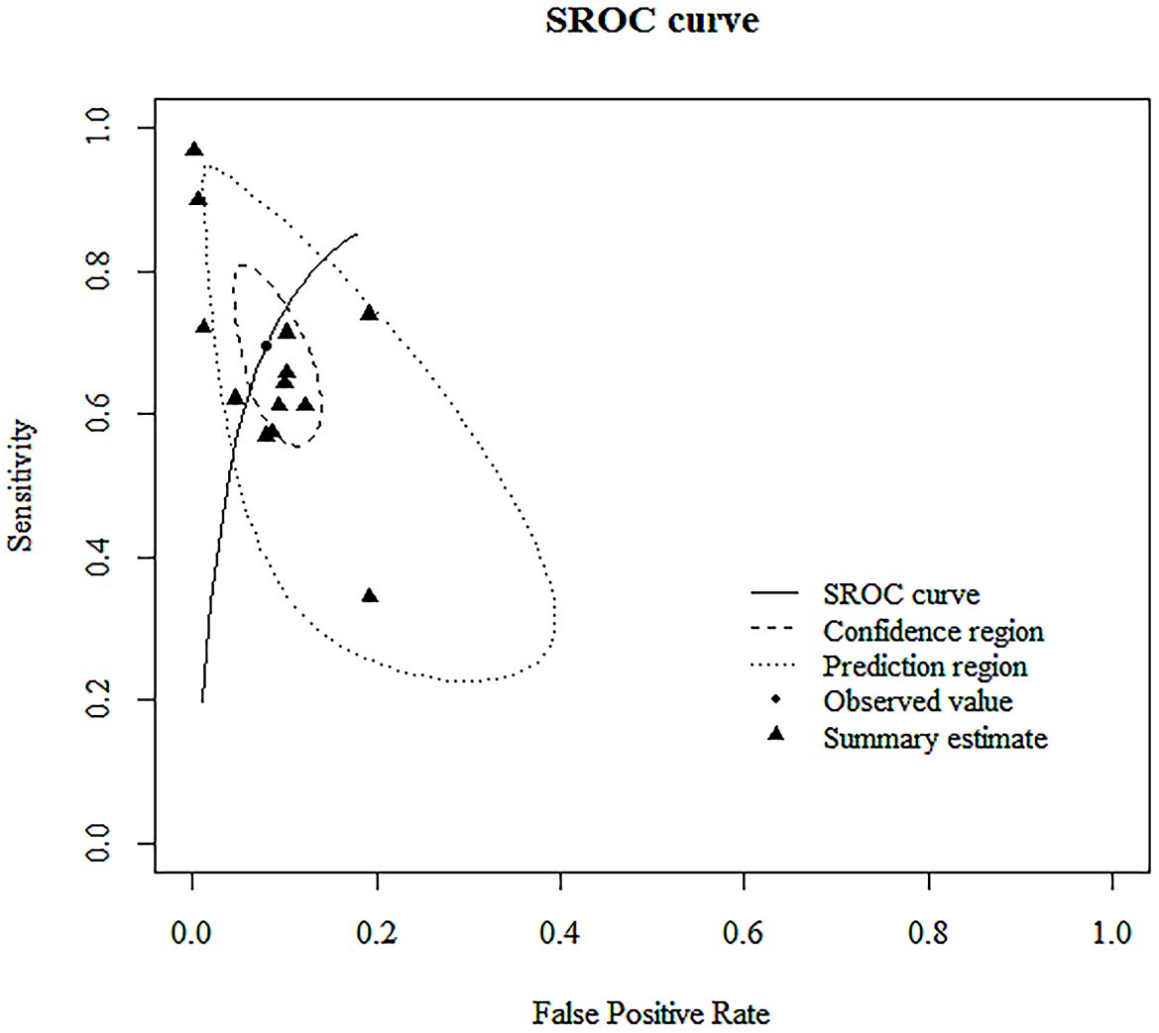
Summary receiver operating characteristic (SROC) curves of the diagnostic accuracy of immunochromatographic tests (ICTs) for *Mycoplasma pneumoniae* infection. Summary points of the sensitivity and specificity, SROC curve, 95% confidence region, and 95% prediction region are shown. The area under the curve of the SROC curve for ICT was 0.904.

The overall sensitivity of the studies included in the analysis was estimated from the bivariate random effects model to be 0.70 (95% CI; 0.59–0.79). Similarly, the overall specificity was estimated to be 0.92 (95% CI; 0.87–0.95). DOR, as shown in Table 2, was 29.20 (95% CI; 10.70–64.20). The AUC for the SROC was 0.904 (Fig 4).

**Table 2:** Summary estimates of the diagnostic accuracy of immunochromatographic tests used to diagnose *Mycoplasma pneumoniae*.

References (year and author)	Sensitivity (95% CI)	Specificity (95% CI)	+LR (95% CI)	−LR (95% CI)	DOR (95% CI)
**2015 Li**	0.97 (0.90–0.99)	1.00 (0.98–1.00)	435.76 (27.33–6947.27)	0.03 (0.01–0.11)	13739.40 (652.36–289,367.43)
**2015 Miyashita**	0.61 (0.31–0.85)	0.91 (0.84–0.95)	6.46 (2.97–14.04)	0.43 (0.19–0.98)	15.04 (3.41–66.29)
**2015 Yamazaki**	0.74 (0.64–0.82)	0.81 (0.73–0.87)	3.86 (2.64–5.63)	0.32 (0.22–0.47)	11.92 (6.21–22.88)
**2016 Miyashita -1**	0.71 (0.57–0.82)	0.90 (0.86–0.92)	6.95 (4.87–9.93)	0.32 (0.20–0.50)	21.72 (10.59–44.57)
**2016 Miyashita -2**	0.61 (0.31–0.85)	0.88 (0.77–0.94)	4.97 (2.13–11.62)	0.44 (0.19–1.01)	11.21 (2.40–52.43)
**2016 Sano -1**	0.57 (0.41–0.72)	0.91 (0.86–0.95)	6.61 (3.61–12.09)	0.47 (0.32–0.69)	14.15 (5.79–34.57)
**2016 Sano -2**	0.57 (0.41–0.72)	0.92 (0.86–0.95)	7.03 (3.77–13.11)	0.47 (0.32–0.68)	15.01 (6.14–36.68)
**2017 Kakuya -1**	0.34 (0.16–0.59)	0.81 (0.67–0.90)	1.78 (0.72–4.41)	0.81 (0.55–1.19)	2.19 (0.61–7.83)
**2017 Kakuya -2**	0.66 (0.41–0.84)	0.90 (0.77–0.96)	6.42 (2.50–16.50)	0.38 (0.19–0.76)	16.76 (4.05–69.30)
**2017 Song**	0.72 (0.62–0.80)	0.99 (0.89–1.00)	58.99 (3.74–929.82)	0.28 (0.21–0.39)	207.65 (12.33–3,495.88)
**2018 Namkoong -1**	0.90 (0.81–0.95)	0.99 (0.95–1.00)	152.77 (9.62–2,425.12)	0.10 (0.05–0.20)	1498.47 (84.06–26,712.36)
**2018 Namkoong -2**	0.64 (0.53–0.74)	0.90 (0.82–0.95)	6.42 (3.32–12.42)	0.40 (0.29–0.54)	16.13 (6.87–37.87)
**2019 Yang**	0.62 (0.53–0.70)	0.95 (0.89–0.98)	13.38 (5.37–33.35)	0.40 (0.31–0.50)	33.66 (12.19–92.92)
**Summary estimates**	**0.70 (0.59**–**0.79)**	**0.92 (0.87**–**0.95)**	**8.94 (4.90**–**14.80)**	**0.33 (0.22**–**0.46)**	**29.20 (10.70**–**64.20)**

CI, confidence interval; +LR, positive likelihood ratio, −LR, negative likelihood ratio, DOR, diagnostic odds ratio

### Subgroup analysis

According to our covariate significance test using a bivariate random effects model, the index test assay was the only significant heterogeneity factor (S3 Table). However, because subgroup analysis may be valuable based on the clinical characteristics, we conducted subgroup analysis to identify each potential source of heterogeneity. The summary estimates for the different subgroups are presented in Table 3.

**Table 3:** Subgroup analyses: Summary estimates using a bivariate random effects model.

Variables	Sensitivity	Specificity	+LR	−LR	DOR	AUC^§^
**Population**						
Children* (n = 6)	0.72 (0.49–0.87)	0.94 (0.80–0.98)	15.40 (2.93–45.20)	0.32 (0.13–0.59)	70.80 (5.19–285.00)	0.911
**Mixed (children + adult) (n = 7)**	0.68 (0.56–0.78)	0.91 (0.88–0.92)	7.12 (5.48–8.98)	0.36 (0.24–0.49)	20.80 (11.60–34.50)	0.906
**Type of specimen**						
**Nasopharyngeal swab (n = 5)**	0.64 (0.48–0.77)	0.87 (0.82–0.91)	4.92 (3.24–7.03)	0.42 (0.26–0.60)	12.50 (5.57–24.50)	0.866
**Oropharyngeal swab (n = 4)**	0.74 (0.58–0.86)	0.96 (0.84–0.99)	21.50 (3.98–64.80)	0.29 (0.15–0.47)	98.60 (8.84–371.00)	0.907
**Reference standard**						
**PCR (n = 11)**	0.72 (0.59–0.82)	0.92 (0.86–0.95)	8.88 (4.52–15.10)	0.31 (0.20–0.46)	31.80 (10.50–73.20)	0.908
**Culture (n = 2)**	0.61 (0.52–0.69)	0.94 (0.89–0.97)	10.20 (5.13–18.30)	0.43 (0.34–0.52)	24.70 (10.70–46.90)	0.763
**Index test assay**						
Ribotest Mycoplasma^®^ (n = 8)	0.66 (0.60–0.71)	0.89 (0.85–0.92)	6.00 (4.55–7.88)	0.39 (0.33–0.45)	15.70 (11.00–21.40)	0.786
**Others (n = 5)**	0.79 (0.55–0.92)	0.98 (0.91–1.00)	49.20 (6.61–157.00)	0.23 (0.08–0.47)	378.00 (14.40–1750.00)	0.962

Numbers are pooled estimates with 95% confidence intervals (CI) in parentheses. Horizontal lines indicate 95% CIs.

^+^LR, positive likelihood ratio,

^−^LR, negative likelihood ratio, DOR, diagnostic odds ratio; AUC, area under the summary receiver operating characteristic curve.

*Children and adults were defined as younger and older than 18 years of age, respectively.

^§^ The area under the summary receiver operating characteristic curve was obtained from a fitted bivariate random effects model and used to summarize overall test performance.

ICT showed similar pooled sensitivity and specificity in populations of children and mixed populations (children + adults). The specimens obtained from oropharyngeal swabs showed a higher sensitivity and specificity than those from nasopharyngeal swabs. In addition, the pooled estimates of sensitivity and accuracy for studies using PCR reference standards were higher than those using microbial culturing (Table 3).

Among the 13 studies included in our current analysis, eight of the studies consisting of 1,287 samples used the Ribotest Mycoplasma^®^ brand of ICT [30, 36, 47–50]. The pooled sensitivity, specificity, positive LR, negative LR, and DOR of Ribotest Mycoplasma^®^ for *M*. *pneumoniae* infection were 0.66 (95% CI, 0.60–0.71), 0.89 (95% CI, 0.85–0.92), 6.00 (95% CI, 4.55–7.88), 0.39 (95% CI, 0.33–0.45) and 15.70 (95% CI, 11.00–21.40), respectively (S4 Table). The overall accuracy of Ribotest Mycoplasma^®^ was 0.786 (S2 Fig). As only one study provided information regarding blinding prior to testing, we were unable to conduct subgroup analysis.

## Discussion

The current systematic review and meta-analysis is the first to establish an overview of the diagnostic accuracy of ICT for *M*. *pneumoniae* infection. ICT showed high specificity (0.92), with modest sensitivity (0.70) for the diagnosis of *M*. *pneumoniae* infection. The SROC AUC was 0.904, which indicates that ICT was highly accurate in the diagnosis of *M*. *pneumoniae* infection. This means that if a test result was positive, it was unlikely to be a false-positive result [64]. Therefore, physicians can confidently make a diagnosis of *M*. *pneumoniae* infection for a patient with respiratory symptoms and a positive ICT result and can then start proper antibiotic treatment to control the infection. Unfortunately, negative ICT results cannot be used to definitely rule out *M*. *pneumoniae* infection [64]. Therefore, the diagnosis should be confirmed using other laboratory methods if the test result can influence management decisions of patients.

Regardless, the easy-to-perform, rapid, accurate diagnosis of *M*. *pneumoniae* infection using ICT has the potential to decrease disease burden by the early prevention of outbreaks in closed populations, such as schools, colleges, and nursing home [5, 65]. ICT may also be a useful test during epidemic outbreaks, even in environments such as private hospitals that may not have specialized laboratories or emergency rooms required for making quick diagnoses. In addition, *M*. *pneumoniae* infection can cause significant morbidity, and even mortality, in patients of extreme age [5]. Prompt diagnosis and treatment of *M*. *pneumoniae* infection in these patients may be specifically beneficial.

Moreover, the prevalence of macrolide-resistant *M*. *pneumoniae* (MRMP) has recently increased worldwide, reaching prevalence rates up to 80–90%, especially in Asian countries [66–70]. MRMP is associated with severe clinical course (e.g., longer durations of fever, cough, and hospital stays) and more extrapulmonary complications [69, 71]. As macrolides are dramatically less effective against MRMP than against macrolide-sensitive *M*. *pneumoniae*, alternative antibiotic treatment including tetracyclines or fluoroquinolones is warranted in severe cases [12, 70–73]. Although ICT is not able to identify whether a particular strain of *M*. *pneumoniae* is resistant to macrolide or not, until additional genetic testing for MRMP strains become available, rapid *M*. *pneumoniae* diagnosis can provide a clinical basis for the use of alternative antibiotics when no clinical improvement is observed with the use of macrolides as the first line of antibiotics [74].

As for the target age group, our study demonstrated that the diagnostic accuracy of ICT for *M*. *pneumoniae* infection was similar between the groups containing both adults and children and the groups containing only children. This finding suggests that ICT may be used regardless of patient age. Nevertheless, clinical studies evaluating ICT focusing on adult populations and studies that compare the accuracy of ICT between children and adults are still warranted.

The only significant heterogeneity factor in our covariate significance analysis was the index test used. The majority of index tests used in the studies analyzed was the commercially available Ribotest Mycoplasma^®^ kit. Pooled estimates of the sensitivities, specificities, and AUC for Ribotest Mycoplasma^®^ was lower than that of other index tests. The other commercial ICT kits used included the FUJI DRI-CHEM IMMUNO AG Cartridge Myco (Fujifilm, Kanagawa, Japan) and the Quick Chaser^®^ Auto Myco *M*. *pneumoniae* antigen detection kit (Mizuho Medy, Saga, Japan), which uses silver amplification. These have been shown to exhibit high sensitivity and specificity (0.904 and 1.0, respectively), even surpassing Ribotest Mycoplasma^®^ (0.644 and 0.905, respectively) in head-to-head comparisons [36]. Test results derived from commercial kits would be more concern for clinicians in the practical use, but there remains a lack of research evaluating the head-to-head performance of ICTs across commercial brands.

The best sampling site for detecting *M*. *pneumoniae*, whether OP swabs or NP swabs, remains controversial [75–77]. In our meta-analysis, higher sensitivity and specificity were found for OP swabs. However, our findings were limited since head-to-head comparisons were not performed in most of the studies included in our analyses. Only one study reported that using OP swab specimens for ICT analysis showed higher accuracy in detecting lower RTIs caused by of *M*. *pneumoniae* than that of NP swab specimens when the samples were concomitantly obtained [49]. The authors suspected the reasons for their findings was due to varied *M*. *pneumoniae* density in the NP swab specimens, which were collected in a blinded fashion, and the larger OP swab tip, which was able to reach deeper into the airway resulting in a specimen higher load of *M*. *pneumoniae*. In addition, it has been reported that a higher copy number of *M*. *pneumoniae* is found in the alveoli than on the epithelium of the upper respiratory tract [78, 79].

Microbial culturing and PCR are currently the most commonly used reference standards for *M*. *pneumoniae* diagnosis. *M*. *pneumoniae* culturing has specific short-comings, including being less sensitive, difficult to perform, and requiring longer than PCR to obtain results [17, 80–84]. In our current analysis, the overall sensitivity and accuracy of ICT using PCR as the reference standard were higher than of ICT using microbial culturing, but the overall specificity was similar. Accordingly, we suggest that PCR is a more useful reference standard for ICT because of the moderate pooled sensitivity of ICT.

It is noteworthy that our current study had several limitations. First, there were no studies included that assessed the difference in ICT performance between macrolide-sensitive and macrolide-resistant *M*. *pneumoniae* strains. In addition, industrial sponsorship, inclusion/exclusion of comorbid conditions, duration of clinical symptoms prior to testing, time lapse before specimen processing, and blinded assessment of ICT were so rarely reported that we were not able to evaluate their effects.

## Conclusions

ICT is a rapid and easy-to-use detection method with moderate sensitivity, high specificity, and high accuracy in diagnosing *M*. *pneumoniae* infection, regardless of patient age. This suggests that ICT is a useful test during the diagnostic workup of RTIs. Major practical advantages of ICT are its user-friendly format and short time requirements (usually ≤ 20 minutes). ICT could function as the point-of-care in clinical practice, instead of serology, PCR and microbial culturing, especially in resource-limited settings. If physicians are aware of the limitations of ICT, such as false negative results, they could make educated decisions in using ICT to implement appropriate antibiotics stewardship and infection control as well as to help make decision regarding the use of other diagnostic modalities. However, additional studies regarding the potential impact of ICT in clinical practice are necessary. These include the cost-effectiveness of routine ICT use and whether ICT may allow for decreases in additional diagnostic tests and result in reducing the excessive use of macrolides.

## Supporting information

S1 Checklist Preferred Reporting Items for Systematic Reviews and Meta-Analyses (PRISMA) checklist. (DOC)

S1 Search strategy (DOCX)

S1 Table Demographics of the studies included in the current meta-analysis. (DOCX)

S2 Table Quality assessment of enrolled studies using the Quality Assessment of Diagnostic Accuracy Studies 2 (QUADAS-2) tool. (DOCX)

S3 Table Covariate significance test using bivariate random-effect model. (DOCX)

S4 Table Summary estimates of the diagnostic accuracy according to index test assay to diagnose *Mycoplasma pneumoniae*. (DOCX)

S1 Fig Funnel plot of the studies included in the current meta-analysis. (EPS)

S2 Fig Summary receiver operating characteristic (ROC) curves of the diagnostic accuracy of Ribotest Mycoplasma^®^ for *Mycoplasma pneumoniae* infection. Summary points of the sensitivity and specificity, summary receiver operating characteristic (SROC) curve, 95% confidence region, and 95% prediction region are shown. The area under the curve of the SROC for the immunochromatographic tests (ICTs) was 0.786.
(EPS)
