# Supplementary material for: Immunochromatography for the diagnosis of Mycoplasma pneumoniae infection: A systematic review and meta-analysis
Source: PLoS One. 2020 Mar 17;15(3):e0230338. doi: 10.1371/journal.pone.0230338 (PMC7077834; doi:10.1371/journal.pone.0230338)
Supplement: S1 Search strategy — (DOCX) [file pone.0230338.s002.docx]

**Search Strategy**

**< Medline/Pubmed >**

| Search | Query |
| --- | --- |

#9 Search (("Mycoplasma pneumoniae"[Mesh]) OR (Mycoplasma pneumoniae[tiab])) AND (("Chromatography, Affinity"[Mesh]) OR
 (Immunochromatograph*[tiab]) OR (lateral flow test*[tiab]) OR (lateral flow assay[tiab]))

#8 Search ("Chromatography, Affinity"[Mesh]) OR (Immunochromatograph*[tiab]) OR (lateral flow test*[tiab]) OR (lateral flow assay[tiab])

#7 Search ("Mycoplasma pneumoniae"[Mesh]) OR (Mycoplasma pneumoniae[tiab])

#6 Search lateral flow assay[tiab]

#5 Search lateral flow test*[tiab]

#4 Search Immunochromatograph*[tiab]

#3 Search "Chromatography, Affinity"[Mesh]

#2 Search Mycoplasma pneumoniae[tiab]

#1 Search "Mycoplasma pneumoniae"[Mesh]

**< ISI Web of Science >**

| ID | Search |
| --- | --- |
| # 6 | #1 AND #5 |
| # 5 | #2 OR #3 OR #4 |
| # 4 | ALL=(Lateral flow assay) |
| # 3 | ALL=(Lateral flow test*) |
| # 2 | ALL=(Immunochromatograph*) |
| # 1 | ALL=(Mycoplasma pneumoniae) |

**< Embase >**

No. Query

#10 #8 AND #9

#9 #3 OR #4 OR #5 OR #6 OR #7

#8 #1 OR #2

#7 'lateral flow test*':ti,ab

#6 'lateral flow assay':ti,ab

#5 'lateral flow assay'/exp

#4 'immunochromatograph*':ti,ab

#3 'immunochromatography'/exp

#2 'mycoplasma pneumoniae':ti,ab

#1 'mycoplasma pneumoniae'/exp OR 'mycoplasma pneumoniae'

**< Cochrane Library >**

ID Search

#1 MeSH descriptor: [Mycoplasma pneumoniae] explode all trees

#2 MeSH descriptor: [Chromatography, Affinity] explode all trees

#3 (Mycoplasma pneumoniae):ti,ab,kw

#4 (Immunochromatograph*):ti,ab,kw

#5 (Lateral flow test*):ti,ab,kw

#6 (Lateral flow assay):ti,ab,kw

#7 #2 OR #4 OR #5 OR #6

#8 #1 OR #3

#9 #7 AND #8
