# Supplementary material for: Immunochromatography for the diagnosis of Mycoplasma pneumoniae infection: A systematic review and meta-analysis
Source: PLoS One. 2020 Mar 17;15(3):e0230338. doi: 10.1371/journal.pone.0230338 (PMC7077834; doi:10.1371/journal.pone.0230338)
Supplement: S1 Table — (DOCX) [file pone.0230338.s003.docx]

**S1 Table. Demographics of the studies included in the current meta-analysis**

| **References** | **Age** | **Gender** |
| --- | --- | --- |
| **2015 Li** | range: 3 mo to 10 yr | male: 177, females: 125 |
| **2015 Miyashita** | NM (adult and adolescent) | NM |
| **2015 Yamazaki** | mean, 8.6 yr | male: 107, female: 105 |
| **2016 Miyashita-1** | Laboratory-confirmed MP infection patient (mean 36.1yr; range 16-64 yr) No laboratory-confirmed patient (mean 38.6 yr; range 16-56 yr) | male: 169, female:232 |
| **2016 Miyashita-2** | Laboratory-confirmed CAP patient (n=11, mean 33.8 yr), No laboratory-confirmed CAP patient (n=57, NA) | Laboratory-confirmed CAP patient (n=11, male: 5 and female: 6), No laboratory-confirmed CAP patient (n=57, NM) |
| **2016 Sano-1** | children (mean 7.9±4.5 yr; n=137) and adult (mean 45.4±22.0 yr; n=39) | male: 93, female: 83 |
| **2016 Sano-2** | children (mean 7.9±4.5 yr; n=137) and adult (mean 45.4±22.0 yr; n=39) | male: 93, female: 83 |
| **2017 Kakuya-1** | median 8.8 yr (range, 1-17 yr) | male: 36, female: 22 |
| **2017 Kakuya-2** | median 8.8 yr (range, 1-17 yr) | male: 36, female: 22 |
| **2017 Song** | range, 3 to 14 yr | male: 81, females: 56 |
| **2018 Namkoong-1** | children (≤15 yr, n=123) and adults (range, 16–93 yr; n=34) | male: 89, female:68 |
| **2018 Namkoong-2** | children (≤15 yr, n=123) and adults (range, 16–93 yr; n=34) | male: 89, female:68 |
| **2019 Yang** | median, 5 yr | male: 117, female: 98 (male:female=1:0.84) |

CAP, community acquired pneumonia; MP, *Mycoplasma pneumoniae*; NM, not mentioned.

The term “children”, “adolescent” and “adult” were used as described in each included study in this table.

Table 2. **Summary estimates of the diagnostic accuracy of immunochromatographic assays to diagnose Mycoplasma pneumoniae.**

| **References** | **Sensitivity (95% CI)** | **Specificity (95% CI)** | **LR+ (95% CI)** | **LR- (95% CI)** | **DOR (95% CI)** |
| --- | --- | --- | --- | --- | --- |
| 2015 Miyashita | 0.62 (0.31, 0.86) | 0.91 (0.84, 0.95) | 16.67 (3.46, 80.28) | 6.88 (3.09, 15.28) | 0.41 (0.17, 1.01) |
| 2015 Yamazaki | 0.74 (0.64, 0.82) | 0.81 (0.73, 0.87) | 12.29 (6.36, 23.73) | 3.92 (2.68, 5.74) | 0.32 (0.22, 0.46) |
| 2016 Miyashita -1 | 0.72 (0.57, 0.83) | 0.90 (0.86, 0.93) | 22.49 (10.86, 46.60) | 7.07 (4.94, 10.13) | 0.31 (0.20, 0.50) |
| 2016 Miyashita -2 | 0.62 (0.31, 0.86) | 0.88 (0.78, 0.94) | 12.62 (2.46, 64.67) | 5.36 (2.22, 12.90) | 0.42 (0.17, 1.04) |
| 2017 Kakuya -1 | 0.33 (0.15, 0.58) | 0.81 (0.67, 0.90) | 2.19 (0.58, 8.19) | 1.79 (0.69, 4.63) | 0.82 (0.56, 1.20) |
| 2017 Kakuya -2 | 0.67 (0.42, 0.85) | 0.91 (0.78, 0.96) | 19.50 (4.41, 86.27) | 7.17 (2.64, 19.47) | 0.37 (0.18, 0.76) |
| 2018 Namkoong -2 | 0.64 (0.53, 0.74) | 0.90 (0.82, 0.95) | 17.17 (7.18, 41.07) | 6.76 (3.42, 13.36) | 0.39 (0.29, 0.54) |
| 2019 Yang | 0.62 (0.53, 0.70) | 0.96 (0.90, 0.98) | 37.82 (13.01, 109.98) | 14.92 (5.66, 39.35) | 0.39 (0.31, 0.50) |
| **Summary estimates** | **0.66 (0.60, 0.71)** | **0.89 (0.85, 0.92)** | **6.03 (4.53, 7.99)** | **0.39 (0.33, 0.44)** | **15.80 (10.70, 22.20)** |

Table 3. **Summary estimates**

| **References** | **Sensitivity (95% CI)** | **Specificity (95% CI)** | **LR+ (95% CI)** | **LR- (95% CI)** | **DOR (95% CI)** |
| --- | --- | --- | --- | --- | --- |
| 2015 Miyashita | 0.62 (0.31, 0.86) | 0.91 (0.84, 0.95) | 16.67 (3.46, 80.28) | 6.88 (3.09, 15.28) | 0.41 (0.17, 1.01) |
| 2015 Yamazaki | 0.74 (0.64, 0.82) | 0.81 (0.73, 0.87) | 12.29 (6.36, 23.73) | 3.92 (2.68, 5.74) | 0.32 (0.22, 0.46) |
| 2016 Miyashita -1 | 0.72 (0.57, 0.83) | 0.90 (0.86, 0.93) | 22.49 (10.86, 46.60) | 7.07 (4.94, 10.13) | 0.31 (0.20, 0.50) |
| 2016 Miyashita -2 | 0.62 (0.31, 0.86) | 0.88 (0.78, 0.94) | 12.62 (2.46, 64.67) | 5.36 (2.22, 12.90) | 0.42 (0.17, 1.04) |
| 2017 Kakuya -1 | 0.33 (0.15, 0.58) | 0.81 (0.67, 0.90) | 2.19 (0.58, 8.19) | 1.79 (0.69, 4.63) | 0.82 (0.56, 1.20) |
| 2017 Kakuya -2 | 0.67 (0.42, 0.85) | 0.91 (0.78, 0.96) | 19.50 (4.41, 86.27) | 7.17 (2.64, 19.47) | 0.37 (0.18, 0.76) |
| 2018 Namkoong -2 | 0.64 (0.53, 0.74) | 0.90 (0.82, 0.95) | 17.17 (7.18, 41.07) | 6.76 (3.42, 13.36) | 0.39 (0.29, 0.54) |
| 2019 Yang | 0.62 (0.53, 0.70) | 0.96 (0.90, 0.98) | 37.82 (13.01, 109.98) | 14.92 (5.66, 39.35) | 0.39 (0.31, 0.50) |
|  |  |  |  |  |  |
| **Summary estimates** | **0.66 (0.60, 0.71)** | **0.89 (0.85, 0.92)** | **6.03 (4.53, 7.99)** | **0.39 (0.33, 0.44)** | **15.80 (10.70, 22.20)** |

**Suppl. Table 1. Statistical heterogeneity test**

| **Statistical heterogeneity** | **Result** |
| --- | --- |
| For Sensitivity, chi-squared, p-value | Chi-squared=63.75, p-value<.0001 |
| For Specificity, chi-squared, p-value | Chi-squared=60.62, p-value<.0001 |
| For DOR, Q-statistic, p-value | Q-statistic=44.66, p-value<.0001 |
| Correlation between sensitivity and false positive rate | -0.805 |

Suppl. **Table 2. Covariate significance test**

|  | **Estimate (SE)** | **P-value** |
| --- | --- | --- |
| Logit transformed sensitivity | -0.756 (0.49) | 0.119 |
| Logit transformed false positive rate | 1.284 (0.49) | 0.009 |

Suppl. Table 3. **Statistical heterogeneity test for Ribotest Mycoplasma**

| **Statistical heterogeneity** | **Result** |
| --- | --- |
| For Sensitivity, chi-squared, p-value | Chi-squared=11.16, p-value=0.1319 |
| For Specificity, chi-squared, p-value | Chi-squared=16.23, p-value=0.0231 |
| For DOR, Q-statistic, p-value | Q-statistic=12.83, p-value=0.0763 |
