# Supplementary material for: Immunochromatography for the diagnosis of Mycoplasma pneumoniae infection: A systematic review and meta-analysis
Source: PLoS One. 2020 Mar 17;15(3):e0230338. doi: 10.1371/journal.pone.0230338 (PMC7077834; doi:10.1371/journal.pone.0230338)
Supplement: S2 Table — (DOCX) [file pone.0230338.s004.docx]

**S2 Table. Quality assessment of enrolled studies using** **the Quality Assessment of Diagnostic Accuracy Studies 2 (QUADAS-2) tool.**

| **Study** | **RISK OF BIAS** | | | | **APPLICABILITY CONCERNS** | | |
| --- | --- | --- | --- | --- | --- | --- | --- |
|  | **PATIENT SELECTION** | **INDEX TEST** | **REFERENCE STANDARD** | **FLOW AND TIMING** | **PATIENT SELECTION** | **INDEX TEST** | **REFERENCE STANDARD** |
| **2015 Li** | Unclear | Unclear | Low | Low | Unclear | Low | Low |
| **2015 Miyashita** | Low | Unclear | Low | Low | Unclear | Low | Low |
| **2015 Yamazaki** | Unclear | Unclear | Low | Low | Unclear | Low | Low |
| **2016 Miyashita -1** | Unclear | Unclear | Low | Low | Low | Low | Low |
| **2016 Miyashita -2** | Unclear | Unclear | Low | Low | Low | Low | Low |
| **2016 Sano -1** | Unclear | Unclear | Low | Low | Low | Low | Low |
| **2016 Sano -2** | Unclear | Unclear | Low | Low | Low | Low | Low |
| **2017 Kakuya -1** | Low | Unclear | Low | Low | Unclear | Low | Low |
| **2017 Kakuya -2** | Low | Unclear | Low | Low | Unclear | Low | Low |
| **2017 Song** | Unclear | Low | Low | Low | Unclear | Low | Low |
| **2018 Namkoong -1** | Low | Low | Low | Low | Low | Low | Low |
| **2018 Namkoong -2** | Low | Unclear | Low | Low | Low | Low | Low |
| **2019 Yang** | Unclear | Low | Low | Unclear | Unclear | Low | Low |
