# Supplementary material for: Immunochromatography for the diagnosis of Mycoplasma pneumoniae infection: A systematic review and meta-analysis
Source: PLoS One. 2020 Mar 17;15(3):e0230338. doi: 10.1371/journal.pone.0230338 (PMC7077834; doi:10.1371/journal.pone.0230338)
Supplement: S3 Table — (DOCX) [file pone.0230338.s005.docx]

**S3 Table. Covariate significance test using bivariate random-effect model**

|  | **Estimate (SE)** | ***P*-value** |
| --- | --- | --- |
| **Type of specimen** |  |  |
| Logit transformed sensitivity | 0.483 (0.477) | 0.312 |
| Logit transformed false positive rate | -0.773 (0.431) | 0.073 |
| **Reference standard** |  |  |
| Logit transformed sensitivity | -0.579 (0.662) | 0.381 |
| Logit transformed false positive rate | -0.324 (0.679) | 0.633 |
| **Intex test assay** |  |  |
| Logit transformed sensitivity | -0.756 (0.485) | 0.119 |
| Logit transformed false positive rate | 1.284 (0.492) | **0.009^*^** |
| **Population** |  |  |
| Logit transformed sensitivity | -0.194 (0.527) | 0.714 |
| Logit transformed false positive rate | 0.017 (0.555) | 0.975 |

^*^*P*-value of <0.05 indicates significant covariate that explain heterogeneity.
