# Supplementary material for: Immunochromatography for the diagnosis of Mycoplasma pneumoniae infection: A systematic review and meta-analysis
Source: PLoS One. 2020 Mar 17;15(3):e0230338. doi: 10.1371/journal.pone.0230338 (PMC7077834; doi:10.1371/journal.pone.0230338)
Supplement: S4 Table — (DOCX) [file pone.0230338.s006.docx]

**S4 Table. Summary estimates of the diagnostic accuracy according to index test assay to diagnose *Mycoplasma pneumoniae***

| **References** | **Sensitivity (95% CI)** | **Specificity (95% CI)** | **DOR (95% CI)** | **LR+ (95% CI)** | **LR- (95% CI)** |
| --- | --- | --- | --- | --- | --- |
| **Index test assay** |  |  |  |  |  |
| **Ribotest Mycoplasma^®^** |  |  |  |  |  |
| 2015 Miyashita | 0.62 (0.31, 0.86) | 0.91 (0.84, 0.95) | 16.67 (3.46, 80.28) | 6.88 (3.09, 15.28) | 0.41 (0.17, 1.01) |
| 2015 Yamazaki | 0.74 (0.64, 0.82) | 0.81 (0.73, 0.87) | 12.29 (6.36, 23.73) | 3.92 (2.68, 5.74) | 0.32 (0.22, 0.46) |
| 2016 Miyashita -1 | 0.72 (0.57, 0.83) | 0.90 (0.86, 0.93) | 22.49 (10.86, 46.60) | 7.07 (4.94, 10.13) | 0.31 (0.20, 0.50) |
| 2016 Miyashita -2 | 0.62 (0.31, 0.86) | 0.88 (0.78, 0.94) | 12.62 (2.46, 64.67) | 5.36 (2.22, 12.90) | 0.42 (0.17, 1.04) |
| 2017 Kakuya -1 | 0.33 (0.15, 0.58) | 0.81 (0.67, 0.90) | 2.19 (0.58, 8.19) | 1.79 (0.69, 4.63) | 0.82 (0.56, 1.20) |
| 2017 Kakuya -2 | 0.67 (0.42, 0.85) | 0.91 (0.78, 0.96) | 19.50 (4.41, 86.27) | 7.17 (2.64, 19.47) | 0.37 (0.18, 0.76) |
| 2018 Namkoong -2 | 0.64 (0.53, 0.74) | 0.90 (0.82, 0.95) | 17.17 (7.18, 41.07) | 6.76 (3.42, 13.36) | 0.39 (0.29, 0.54) |
| 2019 Yang | 0.62 (0.53, 0.70) | 0.96 (0.90, 0.98) | 37.82 (13.01, 109.98) | 14.92 (5.66, 39.35) | 0.39 (0.31, 0.50) |
| **Summary estimates** | **0.66 (0.60, 0.71)** | **0.89 (0.85, 0.92)** | **15.70 (11.00, 21.40)** | **6.00 (4.55, 7.88)** | **0.39 (0.33, 0.45)** |
| **Others** |  |  |  |  |  |
| 2015 Li | 0.97 (0.90, 0.99) | 1.00 (0.98, 1.00) | 13739.40 (652.36, 289367.43) | 435.76 (27.33, 6947.27) | 0.03 (0.01, 0.11) |
| 2016 Sano -1 | 0.57 (0.41, 0.72) | 0.91 (0.86, 0.95) | 14.15 (5.79, 34.57) | 6.61 (3.61, 12.09) | 0.47 (0.32, 0.69) |
| 2016 Sano -2 | 0.57 (0.41, 0.72) | 0.92 (0.86, 0.95) | 15.01 (6.14, 36.68) | 7.03 (3.77, 13.11) | 0.47 (0.32, 0.68) |
| 2017 Song | 0.72 (0.62, 0.80) | 0.99 (0.89, 1.00) | 207.65 (12.33, 3495.88) | 58.99 (3.74, 929.82) | 0.28 (0.21, 0.39) |
| 2018 Namkoong -1 | 0.90 (0.81, 0.95) | 0.99 (0.95, 1.00) | 1498.47 (84.06, 26712.36) | 152.77 (9.62, 2425.12) | 0.10 (0.05, 0.20) |
| **Summary estimates** | **0.79 (0.55, 0.92)** | **0.98 (0.91, 1.00)** | **378.00 (14.40, 1750.00)** | **49.20 (6.61, 157.00)** | **0.23 (0.08, 0.47)** |

CI, confidence interval; LR+, positive likelihood ratio; LR-, negative likelihood ratio; DOR, diagnostic odds ratio
